# Supplementary material for: Content-rich biological network constructed by mining PubMed abstracts
Source: BMC Bioinformatics. 2004 Oct 8;5:147. doi: 10.1186/1471-2105-5-147 (PMC528731; doi:10.1186/1471-2105-5-147)
Supplement: Additional File 2 — The original results of the above study (non-essential files are deleted to keep the file size under the limit set by BMC bioinformatics). [file 1471-2105-5-147-S2.bz2 › chilibotAdditionalFile2/dip10/450ID10477718E10461/html/CCL5_CCR5.html]

 


 **CCL5** and **CCR5** 
  
Found 460 abstracts in PubMed, retrieved 10.  
 

 What does Google say? 
 PDF only 
| .edu only 

---

**Interactive relationship** (e.g. stimulation, inhibition, etc)

**Inhibitory relationship**- CONCLUSIONS Our results demonstrate that an interaction of HGV E2 with CD81 leads to increased RANTES  [ **CCL5** ]  secretion and decreased  **CCR5**  surface expression.  Ref: 12824783 AIDS, 2003
- The chemotactic response of  **CCR5**  CAM T82P to RANTES  [ **CCL5** ]  was similar to that of  **CCR5**  WT, but  **CCR5**  CAM T82K was dramatically decreased.  Ref: 12837756 J Biol Chem< MedlineTA>J Biol Chem, 2003
**Stimulatory relationship**- Whereas the amplitude of calcium mobilization induced by RANTES  [ **CCL5** ]  stimulation was lower in the  **CCR5**  CAM than the wild type WT receptor, MCP 1 induced a higher signal in the CCR2 CAM than in CCR2 WT.  Ref: 12837756 J Biol Chem< MedlineTA>J Biol Chem, 2003
- Enhanced protein concentrations of RANTES  [ **CCL5** ] , MIP 1a, and MIP 1beta were present in WG lung lesions, indicating redundancy of ligands for  **CCR5**  in affected tissue.  Ref: 12858455 J Rheumatol, 2003
**Neutral relationship**- **CCR5**  is a G protein coupled receptor for RANTES  [ **CCL5** ] , MIP 1a, MIP 1b and MCP 2 that functions as the front line coreceptor for human immunodeficiency virus type 1 infection.  Ref: 12837756 J Biol Chem< MedlineTA>J Biol Chem, 2003
- However, the remaining expression of CCR1 and  **CCR5**  correlated reciprocally with an ongoing unresponsiveness to the CCR1 and  **CCR5**  agonists CCL3 macrophage inflammatory protein 1alpha MIP 1alpha, CCL4 MIP 1beta, and  **CCL5**  regulated on activation, normal T expressed and secreted RANTES  [ **CCL5** ] , all chemokines binding to these two receptors.  Ref: 12885942 J Leukoc Biol, 2003
- This interaction induces a dose dependent release of RANTES  [ **CCL5** ]  and down regulation of  **CCR5**  surface expression with concomitant intra cellular accumulation of  **CCR5**  proteins.  Ref: 12824783 AIDS, 2003
- The present findings suggest that MCP 1, RANTES  [ **CCL5** ] , and IP 10 may participate in the pathogenesis of mercuric chloride induced tubulointerstitial fibrosis with mononuclear cell infiltration, via CCR2, CCR1 or  **CCR5** , and CXCR3, respectively.  Ref: 12834626 Exp Mol Pathol, 2003

**Non-interactive relationship** (e.g. studied together, co-existance, homology, etc.)

- Human Hepatic Stellate Cells express  **CCR5**  and RANTES  [ **CCL5** ]  to induce proliferation and migration.  Ref: 12829440 Am J Physiol Gastrointest Liver Physiol< MedlineTA>Am J Physiol Gastrointest Liver Physiol, 2003
- The biopsies were taken in the first 3 months or from the 9th to the 12th month following transplantation.RESULTS IP 10, Mig, RANTES  [ **CCL5** ] , CXCR3, and  **CCR5**  expression levels were increased in the later versus earlier biopsies P< ; =0.01 despite no change in histologic rejection grade status.CONCLUSION These results demonstrate significantly increased expression of T cell chemoattractants in heart allografts during later rejection when compared with episodes occurring shortly after transplantation.  Ref: 12829909 Transplantation, 2003
- METHODS Lung biopsies from 4 patients that had classical features of WG were examined for protein expression of  **CCR5** , RANTES  [ **CCL5** ] , MIP 1a and MIP 1beta using immunohistochemistry.  Ref: 12858455 J Rheumatol, 2003
- After 2 to 4 weeks, L. amazonensis infected mice had significantly delayed and depressed expression of inflammatory cytokines interleukin 12 IL 12, gamma interferon, IL 1alpha, IL 1beta, CC chemokines CC chemokine ligand 3 CCL3 macrophage inflammatory protein 1alpha MIP 1alpha, CCL4 MIP 1beta,  **CCL5**  RANTES  [ **CCL5** ] , MIP 2, and chemokine receptors CCR1, CCR2,  **CCR5**  in foot tissues and draining lymph nodes compared to the expression in L. major infected controls.  Ref: 12874303 Infect Immun, 2003
- Potential molecular differences between early and late rejection grade > =2 episodes were examined by testing chemokine and receptor gene expression.METHODS Expression of inducible protein IP 10, monokine induced by IFN gamma Mig, interferon inducible T cell alpha chemoattractant I TAC, regulated on activation normal T cell expressed and secreted RANTES  [ **CCL5** ] , and their receptors CXCR3 and  **CCR5**  was tested in 60 endomyocardial biopsies from 24 patients using quantitative Taqman real time polymerase chain reaction PCR.  Ref: 12829909 Transplantation, 2003
- In conclusion, RANTES  [ **CCL5** ]  and  **CCR5**  represent potential mediators of 1 HSC migration and proliferation and 2 a cross talk between HSCs and leukocytes during fibrogenesis.  Ref: 12829440 Am J Physiol Gastrointest Liver Physiol< MedlineTA>Am J Physiol Gastrointest Liver Physiol, 2003
- **CCR5**  D32 and RANTES  [ **CCL5** ]  28 and 401 polymorphisms were determined by either specific primers or direct sequencing.  Ref: 12858455 J Rheumatol, 2003
